# Acceptability, Effectiveness, and Roles of mHealth Applications in Supporting Cancer Pain Self-Management: Integrative Review

**DOI:** 10.2196/53652

**Published:** 2024-07-18

**Authors:** Weizi Wu, Teresa Graziano, Andrew Salner, Ming-Hui Chen, Michelle P Judge, Xiaomei Cong, Wanli Xu

**Affiliations:** 1 School of Nursing University of Connecticut Storrs, CT United States; 2 Hartford HealthCare Cancer Institute Hartford, CT United States; 3 Department of Statistics University of Connecticut Storrs, CT United States; 4 Yale School of Nursing Orange, CT United States

**Keywords:** cancer pain, self-management, mHealth applications, integrative review, cancer survivors

## Abstract

**Background:**

Cancer pain remains highly prevalent and persistent throughout survivorship, and it is crucial to investigate the potential of leveraging the advanced features of mobile health (mHealth) apps to empower individuals to self-manage their pain.

**Objective:**

This review aims to comprehensively understand the acceptability, users’ experiences, and effectiveness of mHealth apps in supporting cancer pain self-management.

**Methods:**

We conducted an integrative review following Souza and Whittemore and Knafl’s 6 review processes. Literature was searched in PubMed, Scopus, CINAHL Plus with Full Text, PsycINFO, and Embase, from 2013 to 2023. Keywords including “cancer patients,” “pain,” “self-management,” “mHealth applications,” and relevant synonyms were used in the search. The Johns Hopkins research evidence appraisal tool was used to evaluate the quality of eligible studies. A narrative synthesis was conducted to analyze the extracted data.

**Results:**

A total of 20 studies were included, with the overall quality rated as high (n=15) to good (n=5). Using mHealth apps to monitor and manage pain was acceptable for most patients with cancer. The internal consistency of the mHealth in measuring pain was 0.96. The reported daily assessment or engagement rate ranged from 61.9% to 76.8%. All mHealth apps were designed for multimodal interventions. Participants generally had positive experiences using pain apps, rating them as enjoyable and user-friendly. In addition, 6 studies reported significant improvements in health outcomes, including enhancement in pain remission (severity and intensity), medication adherence, and a reduced frequency of breakthrough pain. The most frequently highlighted roles of mHealth apps included pain monitoring, tracking, reminders, education facilitation, and support coordination.

**Conclusions:**

mHealth apps are effective and acceptable in supporting pain self-management. They offer a promising multi-model approach for patients to monitor, track, and manage their pain. These findings provide evidence-based insights for leveraging mHealth apps to support cancer pain self-management. More high-quality studies are needed to examine the effectiveness of digital technology–based interventions for cancer pain self-management and to identify the facilitators and barriers to their implementation in real-world practice.

## Introduction

Cancer remains a significant health concern in the United States [1]. The cost of cancer health care use in the United States was US $208.9 billion in 2020 and is expected to rise to US $246 billion by 2030 [2]. With advances in cancer diagnosis and treatment and increased survivorship rates, there is a high prevalence of distressing pain, with a pooled prevalence rate of 40% [3]. Cancer pain can persist for months and even years, significantly eroding the quality of life [4-7]. The American Society of Clinical Oncology Practice guideline advocates patient-driven self-management as the primary pain management strategy [8-10]. However, managing cancer pain is a complex and multifaceted experience that poses numerous challenges for patients. These challenges include fear of opioid addiction, insufficient knowledge or skills, and a lack of health care professional supervision [11,12]. In the rapidly advancing digital technology era, it is worth considering leveraging mHealth apps to support patients with evidence-based resources, addressing these concerns, and empowering them with self-management skills to meet personal and social needs [13,14].

The mobile health (mHealth) app is a promising tool for supporting patients in self-managing pain and improving health outcomes due to its popularity, convenience, accessibility, personalization, and cost containment [15,16]. Studies have shown that mHealth interventions could improve medication adherence, self-management engagement, and health outcomes [17-19]. A mixed methods study suggested that individuals with advanced illnesses could greatly benefit from mHealth monitoring systems, which offer continuous patient assessment and critical symptom review information to optimize health outcomes [20]. According to IQVIA’s digital health trends report in 2021, over 350,000 health apps were available in app stores, comprising 47% of all apps, with an increase of about 250 apps per day [21]. However, a content review in 2020 identified only 119 designed for patients with cancer among the thousands available in major mobile app marketplaces [22]. Moreover, a review conducted in 2017 identified 46 apps geared toward clinicians for palliative care guidelines, advance care planning, pharmaceutical tools, and sharing the latest news and opinions related to palliative care [23]. However, the same research team identified only 25 palliative care apps designed specifically for patients or families [24]. Another systematic review in 2021 found that only 101 out of 1189 apps included symptom-tracking features for patients with cancer [25]. However, research on mHealth in cancer pain management is limited. To date, only one systematic review has been done to evaluate the effectiveness of mHealth in managing cancer pain [26]. Reviews of acceptability and end users’ experiences have not been reported. Therefore, this review aims to bridge the research gap and understand the acceptability, effectiveness, and roles of mHealth apps in supporting cancer pain self-management.

## Methods

### Theoretical Preparation

An integrative review is a broad review method that includes data from various research designs to comprehensively understand a phenomenon or interest [27]. The conducting of an integrative review involves a broad, flexible, and interpretative approach in six key steps [28,29]: (1) formulate purpose or review questions; (2) systematically search and select qualitative studies, quantitative studies, and mixed methods studies using predetermined criteria; (3) perform quality appraisal of included studies; (4) narratively and interpretively analyze and synthesize findings; (5) synthesize key and themes and offer insights into the broad implications of the findings; and (6) disseminate plans of findings to audience with diverse interests and applications.

### Literature Searching Strategies

As indicated in the PRISMA (Preferred Reporting Items for Systematic Reviews and Meta-Analyses) flow diagram, this integrative review identified eligible studies by conducting literature searches in an electronic database and manual reference tracking of eligible articles. With the assistance of librarians, we conducted a literature search using both keywords and controlled vocabulary searches in CINAHL, PubMed, PsycINFO, Scopus, and Embase. Keywords and controlled vocabulary used for the search include “cancer patients” or “oncology patients” or “patients with cancer” or “cancer survivors” AND “mobile application” or “mobile app” or “mHealth app” or “mHealth application” or “eHealth app” or “eHealth application” or mHealth or eHealth or “cellular phone” or “cell phone” or “smartphone” AND “pain self-management” or “pain management” or “pain self-care” or “pain relief” or “pain control” or “pain reduction.” Search strategies and results are detailed in 2 appendices (Multimedia Appendices 1 and 2). Upon identifying the included articles, a manual reference tracking method was implemented to identify any additional eligible studies.

### Study Selection Criteria

Studies were eligible for inclusion if they met the following criteria: (1) original empirical studies using qualitative, quantitative, or mixed methods design; (2) the study population included individuals of all ages diagnosed with any type of cancer; (3) assessing the effectiveness, acceptability, and cancer patients’ experiences of mobile apps in cancer pain assessment and management; (4) primary outcomes focused on pain-related health outcomes, app acceptability, or users’ experience; and (5) English-written, peer-reviewed, full-text articles published between 2013 and 2023.

Studies were excluded if (1) apps were exclusively intended for health professionals and caregivers as end users; (2) patients could not use the app independently for pain management; and (3) apps were used solely for delivering interventions, such as videoconferencing apps.

### Study Selection Procedure

**Figure 1:**
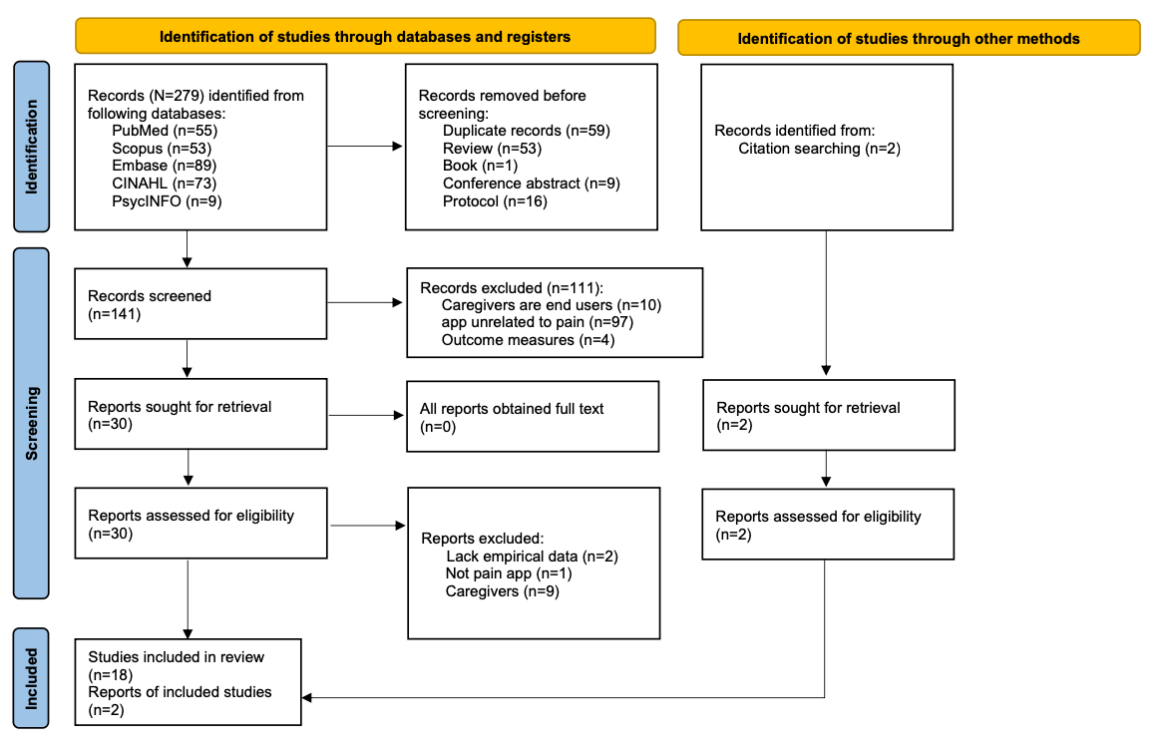
The PRISMA flow diagram. This figure provides the details of (1) identification source (the database searches and reference tracking), (2) the stepwise screening of 281 records (initial screening and abstract, title, and full text), and (3) included 20 records in the review. PRISMA: Preferred Reporting Items for Systematic Reviews and Meta-Analyses.

This integrative review used the PRISMA 2020 flow diagram to report the study selection process (Figure 1). The first author and an experienced librarian conducted the literature search. A total of 279 studies were retrieved by applying the searching strategies as mentioned above. The EndNote program was used to organize the studies and exclude duplicates. Two researchers reviewed the title and abstract of the studies to determine eligibility. The initial screening excluded non-English, nonempirical data, conference abstracts, book documents, reviews, and protocols. Of the 30 remaining papers, 2 researchers did the second round of screening by independently reviewing the full text of each study to determine the eligibility. Discrepancies between reviewers were resolved by discussion and consensus. A total of 18 studies were included after the second-round screening. In addition, 2 studies were identified by tracking the reference lists of included studies, bringing the total number of included articles to 20.

### Quality Assessment

The quality assessment of 20 included studies was conducted using the Johns Hopkins Research Evidence Appraisal Tool, which is commonly used to appraise the qualities of various study designs [30]. The tool consists of distinct checklists to evaluate the quality of quantitative, qualitative, and mixed methods studies. Each checklist includes questions that facilitate determining the quality rated as A, B, or C (high, good, or low quality) and the level of evidence ranging from I (randomized controlled trial [RCT]) to III (nonexperimental-qualitative). Low-quality studies were excluded. Quantitative studies are assessed by a 14-item checklist based on factors including sample size, result consistency, control measures, conclusiveness, and literature review depth. A low-quality quantitative study refers to little evidence with inconsistent results, an insufficient sample size for the study design, and the inability to draw meaningful conclusions. Qualitative studies are assessed using a 13-item checklist emphasizing transparency, diligence, verification, self-reflection, participant-driven inquiry, and insightful interpretation. Low-quality qualitative studies exhibit a lack of clarity and coherence in reporting, lack of transparency in reporting methods, poor interpretation of data, and offer little insight into the phenomena of interest. Mixed methods studies require separate appraisals of both the quantitative and qualitative components and how well the design addresses the research questions. Low quality in mixed methods studies refers to good to low quality of separate quantitative and qualitative components, low relevance of study design, poor levels of integration of data or results, and no consideration of limits of integration. The first author and corresponding author conducted quality assessments and ratings.

### Data Extraction and Synthesis

The authors conducted a comprehensive and iterative review of the included studies to extract overarching findings, and the results were reported following the PRISMA guideline (Multimedia Appendix 3). Table 1 presents the characteristics of the included studies. Due to the heterogeneity of the included studies, a narrative content analysis was conducted to analyze the extracted quantitative and qualitative data. The synthesis captured the patients’ acceptability of mHealth apps, the effectiveness of the targeted outcomes, the features of the apps from the patient’s perspective, and how these features are achieved in the apps (Table 2).

**Table 1:** Basic information of each included study.

Study	Country	Cancer pain context	Cancer type	Age group (years)	Sample size, n
Yang et al [31]	China	Breakthrough pain	General	I^a^: mean 51.10 (SD 8.98), C^b^: mean 53.96 (SD 8.58)	58
Wilkie et al [32]	United States	General	General	Mean 68.4 (SD 14)	234
Hunter et al [33]	United States	General	General, with ALL^c^ (71%)	I: mean 12.25 (SD 3.58), C: mean 11.86 (SD 3.44)	48
Jibb et al [34]	United States	General	General	Mean 14.2 (SD 1.7)	40
Stinson et al [35]	United States	General	General	S1^d^: mean 13.1 (SD 2.9), S2: mean 14.8 (SD 2.8)	106
Villegas et al [36]	Spain	Breakthrough pain	General	Mean 56.95 (SD 10.53)	21
Oldenmenger et al [37]	Netherlands	General	General	Mean 59 (SD 11, range 25-76)	84
Tiozzo et al [38]	Italy	General	Hematologic or solid tumors	Mean 9.1 (SD 5.4, range 0-21)	124
Fu et al [39]	United States	Lymphedema-related pain	Breast cancer	Mean 56.7 (SD 10.6)	120
Lin et al [40]	China	Oral pain	Head and neck cancer	I: mean 49.29 (SD 11.53), C: mean 50.03 (SD 9.21)	64
Salmani et al [41]	Iran	Abdominal pain	Colorectal cancer	Mean 57.18 (SD 17.47)	17
Jibb et al [42]	United States	General	General	Range 12-17	20
Hochstenbach et al [43]	Netherlands	General	General	Mean 53 (SD 15)	11
Bernier Carney et al [44]	United States	Chemotherapy-related pain	General	Median 8 (IQR 6-12)	19
Simon et al [45]	Netherlands	Chemotherapy-related pain	General	Mean 7.33 (SD 5)	27
Jibb et al [46]	United States	General	General	Mean 14.8 (SD 2.1)	16
Fortier et al [47]	United States	General	General	Mean 12.33 (SD 3.42)	12
Azizoddin et al [48]	United States	Advanced pain	Advanced cancer	Adults	14
Alberts et al [49]	Canada	General	Survivors of childhood cancer	I: mean 43.1 (SD 6.9), C: mean 45.0 (SD 10.1)	87
Stinson et al [50]	United States	General	General	S1: mean 13.9 (SD 1.9), S2: mean 13.4 (SD 2.9), S3: mean 13.2 (SD 2.3)	47

^a^I: intervention.

^b^C: control.

^c^ALL: acute lymphoblastic leukemia.

^d^S: study.

**Table 2:** Methodology and main findings of included studies.

Study	Study design	Outcome measures	Findings	Appraisal	
				L^a^	Q^b^
Yang et al [31]	RCT^c^	Primary: app effectiveness Secondary: feasibility	Pain remission Breakthrough pain and adverse reactions reduced QoL^d^ improved Patients were satisfied	I	A
Wilkie et al [32]	Stepped-wedge RCT	Primary: pain intensity and analgesic adherence Secondary: pain misconception	The intervention effect was not significant. 62% of patients viewed the videos on pain misconception	I	A
Hunter et al [38]	Quant^e^	Daily pain assessment Pain intensity	61.2% completed daily dairy Reports of daily average pain were not significant, but fewer moderate to severe pain	III	A
Jibb et al [35]	QED^f^	Primary: feasibility test Secondary: pain intensity, interference, and QoL	Trends in improvements in pain intensity, pain interference, and QoL Mean adherence to pain reporting was 68.8 (SD 38.1%)	II	A
Stinson et al [39]	Quant	Construct reliability and validity	The correlation between pain reports on the app and recall was moderate to high (0.43-0.68) The app’s internal consistency is 0.96	III	A
Villegas et al [36]	QED	Daily pain assessment Usability (System Usability Scale)	Adherence: 61.9% (n=13/21) used the app daily during the 30-day study Breakthrough pain was less frequent Usability: the mean score of System Usability Scale was 85.77/100 (SD 12.09)	II	A
Oldenmenger et al [40]	Quant	Feasibility in a pain diary, pain education, and eConsult	Pain intensity decreased Patients completed the diary for at least 65% of the days Monitoring of pain via the Internet is feasible	III	A
Tiozzo et al [41]	Quant	Pain intensity and characteristics App usage satisfaction	Significant pain relief 94 (75.8%) reported pain at least once per month Most patients were satisfied with the app	III	A
Fu et al [33]	RCT	Pain reduction and QoL	Significant benefits for breast cancer to manage chronic pain soreness, arm and hand swelling, heaviness, and impaired limb mobility	I	A
Lin et al [37]	QED	Pain level and EORCT-QoL	Significant pain relief in the app group at T2 and T3 and significantly higher QoL at T3^g^	II	A
Salmani et al [42]	Quant	Usability evaluation	Average score: 8.03 out of 9 Overall reaction: 7.94 Screen design and layout: 8.18 Systems information: 7.97 Learnability: 7.98 System feature: 8.12	III	B
Jibb et al [43]	Qual^h^	Perceptions of adolescent acceptability, satisfaction, and suggestions for improvement	Enjoy using app Endorse pain advice Facilitate communication with providers Therapeutic benefit Improved awareness of pain	III	A
Hochstenbach et al [46]	MMR^i^	App feasibility and patients’ experience	Learnability (4.8/5), usability (4.8/5), and desirability (4.6/5) Patients were pleased with the simplicity and different components	III	B
Bernier Carney et al [50]	MMR	Pain severity and distress Qualitative pain descriptions	Children are willing to describe their ambulatory pain experiences on a game-based mobile app through quantitative reports and qualitative description	III	B
Simon et al [47]	MMR	App adherence and feasibility Barriers and facilitators of implementation	63% (N=17) used daily for 3 weeks Three facilitators: technical functioning, impact on pain care, and user-friendliness of the app Three barriers: technical problems with daily reminders, content and functionalities, and user-friendliness	III	B
Jibb et al [45]	Qual	Efficiency Ease of use and understanding Utility Acceptability Usability	The time to complete the pain assessment was 4.3 minutes Easy to use and understand Endorse design gamification and customizability Valued content and navigation	III	A
Fortier et al [48]	MMR	Content and usability Patients’ satisfaction	Highly satisfied with the program The 3D Avatar design was attractive The skills training was useful	III	A
Azizoddin et al [44]	Qual	Review wireframes of the content and its delivery	Primary themes: (1) clarity, (2) visual appeal, (3) usefulness, and (4) engagement	III	A
Alberts et al [34]	RCT	Feasibility and acceptability Pain intensity and interference	90.3% (n=28) wore the device >50% of the trial (mean 21.8/30 days [SD 5.9]). 74.2% (23/31) were satisfied with the device. Average pain relieved but not significant. Facilitators: easy to use, beneficial, learning new ways, increased awareness, appreciation	I	A
Stinson et al [49]	MMR	Usability, feasibility, compliance, and satisfaction	Appealing to adolescents Endorsed game-based and virtual reward systems High compliance Likable, easy to use, not bothersome	III	A

^a^L: the level of evidence ranging from I (randomized controlled trial) to III (nonexperimental-qualitative).

^b^Q: quality rated as A, B, or C (high, good, or low quality).

^c^RCT: randomized controlled trials.

^d^QoL: quality of life.

^e^Quant: quantitative.

^f^QED: quasi-experimental design.

^g^The researcher collected data at four time points: before treatment (T0), and the second week (T1), the first month (T2), and the second month (T3) after the start of treatment.

^h^Qual: qualitative.

^i^MMR: mixed methods research.

## Results

### Included Studies Characteristics

The characteristics and methodology of the 20 studies were detailed in Tables 1 and 2. The included studies involved quantitative design (RCT (n=4) [31,32,39,49], quasi-experimental (n=3) [34,36,40], prospective (observational, cohort; n=4) [33,35,37,38], cross-sectional design (n=1) [41], qualitative design (n=3) [42,46,48], and mixed methods design (n=5) [43-45,47,50]). The sample sizes ranged from 11 to 234. A total of 12 studies were conducted in North America (US [n=11] and Canada [n=1]), Europe (the Netherlands [n=3], Spain [n=1], and Italy [n=1]), and Asia (China [n=2] and Iran [n=1]).

#### Quality of Studies

In total, 20 studies included were assessed based on the evidence level and overall quality. Table 2 shows that 4 studies were rated as IA, 3 as IIA, and 8 as IIIA. All of these studies were considered high quality despite different study designs (levels of evidence). In addition, 5 studies were rated as IIIB, which were deemed to be of good quality. No studies were rated as low quality.

#### Pain Context

As shown in Table 1, 6 studies were conducted to assess pain management experiences in specific cancer subgroups or among patients with similar pain subcategories, such as management of oral pain in head and neck cancer [40], abdominal pain in colorectal cancer [41], lymphedema-related chronic pain in breast cancer [39], advanced cancer pain [48], and breakthrough pain [31,36]. In addition, 8 studies were conducted in specific age subgroups other than adult patients with cancer. These included studies involving children [33,38,45,47], school-age children [44], adolescents [34,42], and adult survivors of childhood cancer [49]. Four studies investigated pain management under specific cancer treatment, such as chemotherapy [44,45,47] or concurrent radiotherapy [40].

#### App Context

All pain management apps were developed by multidisciplinary teams that consisted of medical oncologists, palliative care nurse specialists, researchers, and app developers. These apps were created in one of 3 formats, that are (1) dedicated pain management apps (n=7), (2) pain management as the primary module in a comprehensive self-care app (n=5), and (3) pain management integrated into an existing app (n=2). For instance, “Pain Buddy” is a dedicated cancer pain app that helps children with pain self-management [33,47]. “ColorectAlong” is a comprehensive self-care app that includes pain management as one of its 8 components [41]. In addition, Villegas et al [36] detailed the integration of pain management features into an existing app.

### App Feasibility

Overall, 18 studies assessed the feasibility of using mHealth apps in cancer pain management by evaluating usability, acceptability, fidelity, learnability, satisfaction, and desirability. Results indicated that the real-time pain assessment was efficient, valid, and reliable [33,35-38,42,43,45,46,49,50]. One study reported that the average time to complete the pain assessment was 4.3 (SD 3.5) minutes [46]. Another study observed a moderate to vigorous (0.43-0.68) correlation between weekly pain average ratings recorded on the app and retrospective weekly average pain ratings, indicating a high level of internal consistency over 2 weeks (standard Cronbach α=0.96) [35]. Using mHealth apps to monitor or manage pain was acceptable for most patients with cancer [33,37,38,43,45,49]. In total, 5 studies reported adherence to pain assessment during the trial periods (ranging from 2 weeks to 1 month) and found that 61.9%-76.8% of participants completed daily pain assessment. The remaining participants used the app for a shorter period but for at least half of the trial days (minimum 7 days) [33,37,38,43,45]. One study reported that 81% of participants wore the wearable device throughout the 30-day trial period [49]. Another study showed that 18 participants (45%) continued to complete pain assessments and receive treatment advice beyond the study trial [33]. The overall satisfaction with the apps was high, with an average score of 8.9 out of 10 [38], 8.0 out of 9 [41], 4.8 out of 5 [43], and 85.8 out of 100 [36]. Qualitative studies indicated participants’ positive experiences with pain apps, such as likable, enjoyable, easy to use, and not bothersome to complete [42,50].

It is really appealing to the eye. The color, the theme, and the font are good. And it's not really that hard to understand. The vocabulary is really straightforward, and all of the things are on it. The multiple-choice questions and the [visual analog scale] sliders are really easy to use.

42

### Effectiveness of mHealth Apps on Cancer Pain Self-Management

A total of 17 studies demonstrated the effectiveness of the mHealth app in supporting pain outcomes and self-management. Of the 9 studies evaluating the efficacy of pain-related outcomes, 6 reported significant effects of mHealth apps on pain remission (pain severity and pain intensity), improvement in pain medication adherence, reduction of adverse reaction and occurrence of breakthrough pain, and improvement in quality of life [31,34,36,37,39,40]. Two studies observed patterns of pain reduction, although not statistically significant (*P*>.05) [32,49]. In contrast, 1 study showed no significant difference in average daily pain reduction between the intervention and control groups but noted fewer instances of moderate to vigorous pain in the intervention group [33].

In addition, 8 studies included qualitative data from users’ perspectives on cancer pain self-management apps and detailed descriptions of the app. Overall, patients highly valued and emphasized the significant roles of mHealth apps in their daily pain management. A content analysis was conducted to comprehensively understand users’ perspectives and how these app features were implemented. The 3 primary features of pain apps in assisting patients with cancer pain self-management were identified and summarized as (1) pain monitoring, tracking, and reminder, (2) pain education facilitation, (3) pain support coordination.

#### Pain Monitor, Tracker, and Reminder

The primary benefit for patients using mobile apps was monitoring, tracking, and reminding them of their pain management. These apps measured patients’ pain in their daily lives and provided continuous real-time monitoring of pain trends, enabling patients to adjust their medication and management plan [42,47].

It is helpful when the physician calls. I look at the graph to get a good picture. It also gives justification that I'm not exaggerating my pain

43

Pain apps offer various features to assist patients in managing their pain. Three apps provided daily reminders and precisely measured and recorded the cause, severity, intensity, location, nature, type, and duration of pain and the frequency of breakthrough pain, medication taking, and adverse reactions [31,37,40]. The apps used various instruments to assess pain, including the numerical rating scale (NRS) [31,37,45], the Ospedale Pediatrico Bambino Gesù tool [38], the visual analog scale [40,45], the pain diary [37], effective pain descriptors [38], free-text responses [44], and body maps for pinpoint location of pain [31,44,46]. In addition, the app Pain Buddy directed children to pinpoint the pain on an avatar and included a drawing feature to specify the location of the pain in more detail [44].

#### Pain Education Facilitator

The second common function of the apps was to provide pain education. Patients noted that pain educational modules in these apps improved their awareness and interest in pain management. There were 2 studies that reported 100% of users actively engaging in the learning module [42,43].

I thought the pain help ideas were awesome. They would suggest activities like relaxation and breathing. And when you click on it and, there is someone talking to you, walking you through it. Like how to relax

42

Most pain apps incorporate psychoeducational modules that focus on promoting comprehensive pain knowledge and self-management skills based on clinical or World Health Organization guidelines [31,32,36,37,39-43,45,47,49]. A total of 3 apps included features to evaluate patients’ misunderstanding of pain and offered customized information concerning the fundamental causes of pain and the appropriate treatment methods [31,32,37]. In addition, 7 apps included features for self-management skill training, such as medicine instructions, music relaxation treatment or acupuncture [31,36,41], cognitive and behavioral skills training [47], breathing exercises [49], and step-by-step lymphatic exercises for patients with breast cancer [39]. Children users also found the skills training helpful, with belly breathing and distraction techniques being their favorite skills [47]. The trial results of the app, which focused on educating users about oral mucositis knowledge and care skills, showed that the group with access to these resources experienced significantly lower pain levels [40].

Notably, pain apps served as a distraction for many app users, as reported by participants who found it “fun to do” and a “positive challenge” to monitor their pain without constantly focusing on their pain [43,48]. The efficacy of these apps in providing distraction stemmed from the primary principles of app design, emphasizing the engagement and enjoyment of users while considering the unique characteristics and needs of different age groups. For instance, children found the design of 3D Avatar more attractive and enjoyable and were motivated to earn “coins” to customize the program further [46,47]. Adolescents, on the other hand, may be more inclined to gamification elements, including role-playing, badge acquisition, and point and leveling system [35,46], and were attracted by the “appealing to the eye” elements, such as color scheme, fonts, and graphics used [35,42].

#### Pain Support Coordinator

Patients reported that pain apps strengthened their partnerships with health care providers [36,41,42,45]. In particular, some apps offered the feature allowing patients to receive direct phone calls from health care providers when they reported experiencing severe pain, and self-management strategies were ineffective [45]. These features permitted direct and effective communication between patients and the health care team and were highly valued among patients [36]. Further, patients found pain apps enabled more efficient conversations with their health care professionals during office visits by providing precise symptom patterns and notes recorded in daily assessments [41,42].

We don't want to call the hospital all the time. With the app, you get the sense that pain is being monitored, and they call us when we report high pain scores. That is very comforting. It gives you the sense that you're being taken care of

45

Most apps (n=8) allow open dyad access, providing both the patient and their health care provider access to the apps [31,32,36,40,42,43,45,47]. In cases where patients reported a high pain score (over 5/10 or NRS≥4) or moderate to vigorous side effects, the system would automatically remind patients to take medication. One hour later, the system automatically reminded patients to reassess pain [31]. If a high pain score (over 5/10 or NRS≥4) was still reported, an email alert or clinical alarm was sent to clinical professionals to notify the uncontrolled pain condition [36,37]. The medical team could contact the patient directly for alarms when necessary [36,42]. In 2 RCTs, patients assigned to the app group with this alarm feature reported significantly lower frequencies of breakthrough pain and higher medication adherence than the control group and were more likely to promptly detect and address pain exacerbation [31,36]. Clinicians also acknowledged that the app improves treatments’ safety, adherence, and effectiveness for managing breakthrough pain [36].

The eConsult module was embedded in several apps, with an email-like format or social network links, facilitating question-and-answer communication between patients and professionals [32,37,40]. Participants who frequently used eConsults found they were beneficial for pain management [37]. For instance, participants could obtain assistance from this module in setting pain management goals, devising action plans, and identifying in-time coping strategies for breakthrough pain when patients reported high scores [31,36,41]. Outpatients, in particular, appreciated the app’s therapeutic benefit, as it allowed them to receive real-time support and efficient pain management advice without being constrained by time and space limitations [42,48].

It’s a fantastic idea. As one who was living in constant pain, I was not the one to call the doctor. If I had this resource available. Things maybe would have changed for me a lot faster than they (doctors) did

48

## Discussion

### Principal Findings

This integrative review synthesizes the current evidence of mHealth apps supporting pain outcomes and self-management of patients with cancer. Overall, mHealth apps offer significant benefits for managing cancer pain and serve as multi-model interventions that provide critical features such as monitoring, tracking, reminders, education facilitation, and support coordination. These findings offer evidence-based insights into effectively leveraging the advantage of mHealth apps in supporting the pain self-management of patients with cancer.

### Implications

mHealth apps are effective and acceptable in alleviating pain and supporting self-management, as indicated by participants’ feedback and feasibility data. This finding added complementary evidence to systematic reviews that focused exclusively on experimental studies [24,51]. However, integrating these apps into standard clinical care components, such as electronic health records, has posed significant challenges due to factors such as inconsistent app availability [51], standard application programming interface development and adoption in the early stages [52], and security concerns [53]. Despite these challenges, nursing, medical scientists, and other stakeholders have been urged to direct their efforts toward creating a long-term strategic plan for developing and implementing eHealth services, and promoting equitable, accessible, and affordable health care [54]. With the outbreak of COVID-19, videoconferencing apps such as Skype, Zoom, Facebook, 2-way text messages, and other online platforms have played a critical role in transitioning offline health care services to an online format [55]. For example, the electronic patient-reported outcomes (ePROs) system significantly improved health care efficiency, enhanced patient-doctor interaction, and optimized medical resource use [56,57]. Cancer self-management apps have the potential to provide easy access to cancer health care services by eliminating time and space limitations, giving patients greater autonomy and control, and providing more precise or personalized strategies [58]. Further studies are necessary to explore the integration of ePROs into mHealth apps for managing cancer pain.

mHealth apps were promising tools to empower patients with cancer with the necessary knowledge and skills and enable them to manage their pain actively. Patients endorsed and mainly engaged with the educational modules, training programs, and coping strategies offered by pain apps. This finding was consistent with previous studies that patients expressed their educational needs in areas of opioid analgesics, long-term survivorship, and relying on professional service or guidance rather than nonprofessional sources like nonprofessional internet pages or television programs [59,60]. Compared with traditional paper-format educational material, mHealth apps lower the learning threshold and burden while increasing engagement through numerous attractive features such as straightforward, enjoyable, informative, accessible, and personalized designs [61]. Apps can also customize education modules based on patient’s health literacy and ability to use the app over time with the advancement of artificial intelligence and big data [58]. Also, mHealth apps have emerged as a promising tool for translating evidence-based practice into the home setting. The included apps in this review reported that all educational modules were developed based on World Health Organization or clinical or evidence-based guidelines. This approach promotes science communication to the public and facilitates more informed decision-making [62].

mHealth apps could also serve as a distraction technique, drawing patients’ attention away from the mental processing of pain through attractive features. Patients always viewed their cancer journey as life trauma, especially when accompanied by long-term unpleasant pain experiences [63]. Distraction, a simple nonpharmacological technique, is increasingly being examined as an effective intervention. It is applied primarily in pediatric oncological procedure pain [64], during needle insertion or lumbar puncture [65], or subcutaneous port access [66]. With the advancement of digital technology, virtual reality distraction has shown promise in reducing self-reported pain in patients with breast cancer [67], as well as children and adolescents with cancer [66]. Therefore, the app design should fully consider incorporating engagement techniques, that cater to diverse end users’ characteristics, and pain care demands.

mHealth apps had the potential to bridge gaps in health care access and facilitate interaction and communication with health care providers. Previous studies highlighted patients’ challenges in accessing professional survivorship care after completing clinical treatment [68,69]. mHealth app serves as an eConsult medical chatbot, allowing patients to seek professional consultation to address daily minor issues or confusion, thus minimizing unnecessary clinic visits [70]. In addition, mHealth apps with alert systems can help identify urgent situations that require immediate health intervention. Implementing ePROs within clinical practice enables remote monitoring and early detection of severe and worsening symptoms [71]. One clinical randomized trial illustrated that web-based ePRO tools are feasible and acceptable among patients with advanced cancer without increasing clinical burden [72]. However, it would be valuable to explore clinical staff’s perspectives on whether these applications increase or reduce their workload. Furthermore, with the tracking function of pain apps, patients can precisely describe the trends and characteristics of their pain, enhancing the quality of communication and problem-solving efficiency between patients and professionals in pain management. The findings from another systematic review also supported the idea that practical technology tools can help strengthen communication and partnership between patients and providers [73]. For instance, a 2-way text message strategy has been shown to be effective in improving engagement and adherence in the survivorship management program, although it may require the research team to commit more effort to respond to text messages personally [74,75]. Building effective partnerships with providers is one core skill in promoting successful cancer pain self-management [76]. Further studies are needed to evaluate the cost-effectiveness of such interaction designs within the health care system across different contexts and populations.

### Limitations

Several limitations exist in this review that warrant careful consideration. Small sample sizes and short study durations in the preliminary usability testing may limit the generalization of the study findings. There is a risk of a skewed perception of the intervention’s success or survivor bias, particularly if individuals who did not adhere to long-term trials or lost interest in the app interventions were not adequately represented in the data. Further research is imperative to establish causality and generalizability of the findings. It is important to note that the review primarily focuses on patients’ perspectives. Additional reviews focusing on insights from other stakeholders, such as health care professionals and caregivers, are needed to achieve a more holistic understanding before disseminating findings into clinical practice. The heterogeneity of the included studies, including varied designs, pain measures, and app functionalities, along with limited evidence from RCTs, inherently constrains the level of analysis and evidence. Despite meticulous search efforts, limitations in search strategy, including search terms, databases used, inclusion and exclusion criteria, and the fast-paced development of technology, could lead to unintentionally omitting relevant new apps. Finally, while the review predominantly draws from academic literature, a more comprehensive understanding could be gained by incorporating insights from market app stores and usage reports.

### Conclusion

Overall, mHealth apps are effective and acceptable in supporting the self-management of cancer pain. They offer a promising approach for patients to monitor, track, and manage their pain and receive multimodel interventions to promote pain self-management. These findings provide evidence-based insights for leveraging the features of mHealth apps in supporting cancer pain self-management. More high-quality studies are needed on the effectiveness of digital technology–based interventions for cancer pain self-management and to identify the facilitators and barriers to their implementation in real-world practice.

## Appendix

Multimedia Appendix 1 Searching Strategies.

Multimedia Appendix 2 Details of search database, syntax, and results.

Multimedia Appendix 3 PRISMA (Preferred Reporting Items for Systematic Reviews and Meta-Analyses) checklist.

## Abbreviations

ePRO: electronic patient-reported outcomes
mHealth: mobile health
NRS: numerical rating scale
PRISMA: Preferred Reporting Items for Systematic Reviews and Meta-Analyses
RCT: randomized controlled trial
