# Supplementary material for: Acceptability, Effectiveness, and Roles of mHealth Applications in Supporting Cancer Pain Self-Management: Integrative Review
Source: JMIR Mhealth Uhealth. 2024 Jul 18;12:e53652. doi: 10.2196/53652 (PMC11294773; doi:10.2196/53652)
Supplement: Multimedia Appendix 1 [file mhealth_v12i1e53652_app1.docx]

**Table S1**

|  | Concept #1 (Population)  Cancer | Concept #2  Mobile Apps | Concept #3  Pain |
| --- | --- | --- | --- |
| Keywords | Cancer  “Oncology patients”  “Cancer patients”  “Cancer survivors”  “patient with cancer” | “mobile apps”  “mobile applications"  “Smartphone apps”  mHealth  “mHealth applications”  “mHealth apps”  eHealth  “eHealth application”  “eHealth apps” | pain  “pain management”  “cancer pain”  “pain self-management”  “pain self-care”  “pain control”  “pain reduction”  “pain relief” |
| Controlled Vocabulary *(Emtree / MeSH/ Subject/ Thesaurus)* | Neoplasms [MeSH] [Subject]  “Cancer survivors” [MeSH] [Subject]  “cancer patients” [Subject]  malignant neoplasm [Emtree]  cancer survivor [Emtree]  cancer patient [Emtree] | “mobile applications” [MeSH] [Subject] [Thesaurus]  “cell phone” [MeSH]  “smartphones”[MeSH] [Subject] [Thesaurus]  “cellular phone” [Subject]  mobile application [Emtree]  mobile health application [Emtree]  mobile phone [Emtree]  smartphone [Emtree]  mobile health [Thesaurus]  mobile phone + [Thesaurus] | Pain [MeSH] [Subject] [Emtree] [Thesaurus]  “pain management” [MeSH] [Subject] [Thesaurus]  “cancer pain” [MeSH] [Subject] [Emtree]  Chronic Pain [Thesaurus]  Acute pain [Thesaurus] |
