# Supplementary material for: Acceptability, Effectiveness, and Roles of mHealth Applications in Supporting Cancer Pain Self-Management: Integrative Review
Source: JMIR Mhealth Uhealth. 2024 Jul 18;12:e53652. doi: 10.2196/53652 (PMC11294773; doi:10.2196/53652)
Supplement: Multimedia Appendix 2 [file mhealth_v12i1e53652_app2.docx]

**Table S1**

| Database |  | Search syntax | Citations found |
| --- | --- | --- | --- |
| *1) CINAHL* | 1 | (MM "Cancer Survivors") OR (MM "Cancer Patients") |  |
|  | 2 | (MM "Mobile Applications") OR (MM "Smartphone") OR (MM "Cellular Phone+") |  |
|  | 3 | (MM "Pain Management") OR (MM "Cancer Pain") |  |
|  | 4 | AB ( "cancer patients" or "oncology patients" or "patients with cancer" or "cancer survivors" ) OR TI ( "cancer patients" or "oncology patients" or "patients with cancer" or "cancer survivors" ) |  |
|  | 5 | AB ( "mobile applications" or "mobile apps" or "mHealth apps" or "mHealth applications" or "eHealth apps" or "eHealth application" or mHealth or eHealth ) OR TI ( "mobile applications" or "mobile apps" or "mHealth apps" or "mHealth applications" or "eHealth apps" or "eHealth application" or mHealth or eHealth ) |  |
|  | 6 | AB ( "pain self-management" or "pain management" or "pain self-care" or "pain relief" or "pain control" or "pain reduction" ) OR TI ( "mobile applications" or "mobile apps" or "mHealth apps" or "mHealth applications" or "eHealth apps" or "eHealth application" or mHealth or eHealth ) |  |
|  | 7 | (S1 OR S4) AND (S2 OR S5) AND (S3 OR S6 ) |  |
|  | 8 | (S1 OR S4) AND (S2 OR S5) AND (S3 OR S6 ) **Limiters** - Published Date: 20120101-20231231; English Language; Peer Reviewed; Research Article | **73** |
| *2) PubMed* | 1 | (Neoplasms[MeSH Terms]) OR (cancer survivors[MeSH Terms]) |  |
|  | 2 | ((("cancer patients"[Title/Abstract]) OR ("oncology patients"[Title/Abstract])) OR ("cancer survivors"[Title/Abstract])) OR ("patients with cancer"[Title/Abstract]) |  |
|  | 3 | (("mobile applications"[MeSH Terms]) OR ("cell phone"[MeSH Terms])) OR (smartphone[MeSH Terms]) |  |
|  | 4 | ((((((("mobile apps"[Title/Abstract]) OR ("mobile applications"[Title/Abstract])) OR (mHealth[Title/Abstract])) OR (eHealth[Title/Abstract])) OR ("mHealth apps"[Title/Abstract])) OR ("eHealth apps"[Title/Abstract])) OR ("mHealth applications"[Title/Abstract])) OR ("eHealth applications"[Title/Abstract]) |  |
|  | 5 | (("cancer pain"[MeSH Terms]) OR (pain[MeSH Terms])) OR ("pain management"[MeSH Terms]) |  |
|  | 6 | ((("pain management"[Title/Abstract]) OR ("pain self-management"[Title/Abstract])) OR ("pain self-care"[Title/Abstract])) OR ("cancer pain"[Title/Abstract]) |  |
|  | 7 | (#1 OR #2) AND (#3 OR #4) AND (#5 OR #6) |  |
|  | 8 | (#1 OR #2) AND (#3 OR #4) AND (#5 OR #6) Filters: English, 2012-2023 | 55 |
| 1. *Scopus* | 1 | TITLE-ABS-KEY ( "oncology patients" OR "cancer patients" OR "cancer survivors" OR "patient with cancer" ) |  |
|  | 2 | TITLE-ABS-KEY ( "mobile applications" OR "mobile apps" OR "mhealth apps" OR "mhealth applications" OR "ehealth apps" OR "ehealth application" OR mhealth OR ehealth OR "cellular phone" OR " cell phone" OR "smartphone" ) |  |
|  | 3 | TITLE-ABS-KEY ( "pain self-management" OR "pain management" OR "pain self-care" OR "pain relief" OR "pain control" OR "pain reduction" OR "cancer pain" ) |  |
|  | 4 | #1 AND #2 AND #3 |  |
|  | 5 | #1 AND #2 AND #3 AND PUBYEAR > 2011 AND PUBYEAR < 2024 AND ( LIMIT-TO ( LANGUAGE , "English" ) ) | 53 |
| *4) Embase* | 1 | 'mobile application'/exp OR 'mobile health application'/exp OR 'mobile phone'/exp OR 'smartphone'/exp |  |
|  | 2 | 'pain'/exp OR 'cancer pain'/exp |  |
|  | 3 | 'cancer patient'/exp OR 'cancer survivor'/exp |  |
|  | 4 | 'cancer patients':ab,kw,ti OR 'cancer survivors':ab,ti,kw OR 'oncology patients':ab,ti,kw OR 'patients with cancer':ab,kw,ti |  |
|  | 5 | 'pain management':ab,kw,ti OR 'cancer pain':ab,ti,kw OR 'pain self-management':ab,ti,kw OR 'pain reduction':ab,kw,ti OR 'pain control':ab,ti,kw OR 'pain relief':ab,ti,kw OR 'pain self-care':ab,kw,ti |  |
|  | 6 | 'mobile applications':ab,kw,ti OR mhealth:ab,ti,kw OR 'mobile apps':ab,ti,kw OR 'smartphone applications':ab,kw,ti OR 'smartphone apps':ab,ti,kw OR 'mhealth applications':ab,ti,kw OR 'ehealth applications':ab,kw,ti OR ehealth:ab,kw,ti |  |
|  | 7 | (#1 OR #6) AND (#2 OR #5) AND (#3 OR #4) |  |
|  | 8 | #7AND (2012-2023:py) AND ('article'/it OR 'article in press'/it OR 'review'/it) | 89 |
| *5) PsycINFO* | 1 | DE "Mobile Applications" OR DE "Mobile Health" OR DE "Mobile Phones" OR DE "Smartphones" |  |
|  | 2 | DE "Pain" OR DE "Acute Pain" OR DE "Aphagia" OR DE "Back Pain" OR DE "Chronic Pain" OR DE "Headache" OR DE "Myofascial Pain" OR DE "Neuralgia" OR DE "Neuropathic Pain" OR DE "Somatoform Pain Disorder" OR DE "Acute Pain" OR DE "Pain Management" OR DE "Chronic Pain" |  |
|  | 3 | DE "Neoplasms" |  |
|  | 4 | AB ( "cancer patients" or "oncology patients" or "patients with cancer" or "cancer survivors" ) AND TI ( "cancer patients" or "oncology patients" or "patients with cancer" or "cancer survivors" ) |  |
|  | 5 | AB ( "mobile applications" or "mobile apps" or "mHealth apps" or "mHealth applications" or "eHealth apps" or "eHealth application" or mHealth or eHealth ) AND TI ( "mobile applications" or "mobile apps" or "mHealth apps" or "mHealth applications" or "eHealth apps" or "eHealth application" or mHealth or eHealth ) |  |
|  | 6 | AB ( "pain self-management" or "pain management" or "pain self-care" or "pain relief" or "pain control" or "pain reduction" ) AND TI ( "pain self-management" or "pain management" or "pain self-care" or "pain relief" or "pain control" or "pain reduction" ) |  |
|  | 7 | (S3 OR S4) AND (S2 OR S6) AND (S1 OR S5) | 9 |
